# Evaluation of detection performance and cost-effectiveness of IDEXX Colilert-18 and ChromAgar for environmental surveillance of extended-spectrum beta-lactamase *Escherichia coli* in water samples from urban marketplaces in Blantyre, Malawi

**DOI:** 10.1128/spectrum.01695-25

**Published:** 2026-05-20

**Authors:** Effita Fifi Masoamphambe, Jones Chipinga, Madalitso Mphasa, Kondwani Chidziwitsano, Mindy Panulo, James Jafali, Nicholas Feasey, David Berendes, Amy Kirby, Tracy Morse, Derek Cocker

**Affiliations:** 1 Malawi-Liverpool Wellcome Programme, Blantyre, Malawi; 2 Department of Clinical Sciences, Liverpool School of Tropical Medicine 9655, Liverpool, United Kingdom; 3 Centre for Water, Sanitation, Health, and Appropriate Technology Development (WASHTED), Blantyre, Malawi; 4 Malawi University of Business and Applied Sciences 119042, Blantyre, Malawi; 5 School of Medicine, University of St Andrews, St Andrews, United Kingdom; 6 U.S. Centers for Disease Control and Prevention, Atlanta, Georgia, USA; 7 Department of Civil and Environmental Engineering, University of Strathclyde 3527, Glasgow, United Kingdom; 8 David Price Evans Infectious Disease and Global health Group, University of Liverpool 4591, Liverpool, United Kingdom; Commonwealth Scientific and Industrial Research Organization, Brisbane, Australia

**Keywords:** ESBL, *E. coli*, WASH, antimicrobial resistance, environmental surveillance, cost-effectiveness, marketplaces, Malawi

## Abstract

In sub-Saharan Africa, extended-spectrum beta-lactamase (ESBL)-producing bacterial infections pose significant public health risks. The importance of ESBL transmission within the food chain, as well as the role of urban marketplaces in this relationship, is becoming increasingly recognized. ESBL *Escherichia coli* also represents a key indicator organism within global antimicrobial resistance surveillance strategies, including the World Health Organization Tricycle protocol. Therefore, evaluating simple, cost-effective, and scalable detection methods for ESBL-producing *E. coli* is crucial. In 2021, a modified Colilert-18 method and a conventional ChromAgar ESBL culture method were assessed for the detection of ESBL *E. coli* in water samples from four urban marketplaces in Blantyre, Malawi. ESBL *E. coli* were detected in water samples obtained from urban marketplaces using both ChromAgar ESBL culture (39% [*n* = 65/167]) and IDEXX Colilert-18 methods (76% [*n* = 127/167]). Using ChromAgar ESBL as a reference standard, the modified Colilert-18 method showed a sensitivity of 86% and a specificity of 30%. Agreement between the methods was low (Cohen’s kappa = 0.141, *P* < 0.001). The cost per test was lower for Colilert than for ChromAgar, indicating potential cost-effectiveness in primary ESBL *E. coli* detection. At the time of sampling, water collected from urban marketplaces was contaminated with ESBL *E. coli*, posing possible public health risks. Both the ChromAgar method and the modified IDEXX method offer valuable tools for microbiological surveillance of ESBL *E. coli* in urban markets. However, further studies must be conducted to optimize their use and determine additional considerations to factor into cost-effectiveness.

**IMPORTANCE:**

This study highlights the role of urban marketplaces as hotspots for potential transmission of extended-spectrum beta-lactamase (ESBL) bacteria. The findings suggest that markets are critical sites that warrant for environmental surveillance, particularly in settings with limited water, sanitation, and hygiene infrastructure. We investigated the differences in detection performance and the cost-effectiveness of a modified IDEXX Colilert-18 method compared to conventional culture using ChromAgar. We found that the higher detection rate and lower cost of the Colilert-18 method point to its potential as a viable alternative for surveillance of ESBLs in water samples. These findings are particularly relevant for low-income settings, where routine environmental monitoring is constrained due to poor laboratory capacity and limited resources. Our study contributes to the evidence on cost-effective methods for environmental surveillance of antimicrobial-resistant bacteria and emphasizes the need for locally appropriate, scalable tools to support public health responses to antimicrobial resistance.

## OBSERVATION

Antimicrobial resistance (AR) is a global health threat, with the highest burden believed to be in sub-Saharan Africa (1). In Malawi, extended-spectrum beta-lactamase *Escherichia coli* (ESBL-E) is a major concern due to a reliance on the use of third-generation cephalosporins (3-GCs), with ESBL-E infections associated with a high morbidity and mortality (2). The World Health Organization (WHO) emphasizes the significance of environmental surveillance for ESBL-E to understand and address AR, using ESBL-E as a key indicator organism within the WHO Tricycle protocol (3). There is also growing recognition of the importance of risks from water, sanitation, and hygiene (WASH)-related exposures to antimicrobial-resistant bacteria in urban communities, especially via contaminated food chains (3, 4). Within Malawi, there is a paucity of WASH infrastructure alongside high levels of ESBL-E reported in human and animal stool, household produce, and environmental samples (4–6). Establishing efficient, accurate and cost-effective methods for conducting environmental surveillance of extended-spectrum beta-lactamase (ESBL)-producing bacteria in these settings is crucial to understanding and mitigating AR threats.

A range of microbiological methods exist for environmental surveillance of ESBL-E. Previous research has illustrated that a modified IDEXX Colilert assay performs well compared to conventional culture methods for detecting ESBL-E, suggesting the potential for this method for environmental surveillance (7). Within our study, we aimed to compare the ability of a modified IDEXX method to traditional culture on ChromAgar to detect ESBL-producing *E. coli* in diverse water sample types obtained from urban marketplaces. In addition, we compared the costs associated with modified IDEXX Colilert to conventional culture methods (8).

Between February and November 2021, water samples were collected from four urban marketplaces (Ndirande, Majiga, Safarawo, and Ngumbe) in Blantyre, Malawi, at three time points: February–March (rainy season, *n* = 66), May–June (cool-dry season, *n* = 59), and October–November (hot-dry season, *n* = 42). Grab samples (1,000 mL) were obtained from (i) stored water used by market vendors to keep the vegetables fresh during the day; (ii) the primary source of water for the marketplace (i.e., tap/kiosk/borehole); (iii) drain samples at pre-identified risk points which were close to the vegetable selling section of the market; and (iv) water from rivers adjacent to the marketplace. Duplicate water samples were simultaneously processed using the modified IDEXX Colilert-18 and the ChromAgar ESBL culture methods.

The detailed description of the methods used in this document can be accessed online (https://doi.org/10.5281/zenodo.18595969). For the IDEXX Colilert-18 method, the choice of antibiotic was altered from cefotaxime to ceftriaxone due to lack of availability of cefotaxime locally. This substitution constitutes the modified method referred to throughout this study. Data were analyzed using R Studio (R statistical software version 4.2.3). The outcomes of interest for the tests were binary (presence or absence of *E. coli*), allowing for assessment of detection sensitivity. Agreement between the modified IDEXX Colilert-18 method and the ChromAgar ESBL conventional culture was assessed using Cohen’s kappa statistic and McNamar’s test.

In total, 167 water samples were collected at urban marketplaces across the three time points representing rainy, cold, and dry seasons. ChromAgar ESBL and IDEXX Colilert methods detected ESBL-E in 39% (*n* = 65) and 76% (*n* = 127) of samples, respectively, indicating a high presence of ESBL-E in water at these sites (Table 1). Detection by ChromAgar varied significantly across seasons, with the highest positivity observed in the rainy season and the lowest in the cool-dry season (*P* < 0.001), while Colilert showed less pronounced seasonal variation (Table S1). These results are consistent with prior research indicating the presence of ESBL-E in urban environments, water samples, and household foods in southern Malawi (5, 9). Without improved access to and use of WASH facilities and practices in these settings, market water provides an ongoing exposure risk for the transmission of antimicrobial-resistant bacteria. Agreement between ChromAgar and Colilert methods was observed in 52% (*n* = 87) of the samples, showing wide variability. However, secondary testing of Colilert broth with ChromAgar revealed 100% recovery of ESBL-E.

**TABLE 1:** Direct comparison of modified IDEXX Colilert-18 and ChromAgar ESBL culture methods^*a*^

ChromAgar ESBL (reference)	Colilert detected	Colilert not detected	Total
Detected	56	9	65
Not detected	71	31	102
Total	127	40	167

^*a*^ Sensitivity: 86%; specificity: 30%; accuracy: 52%; Cohen’s kappa = 0.141, *P* < 0.001.

The apparent higher detection rate and low cost of IDEXX Colilert-18 compared to ChromAgar ESBL is promising; however, this may hypothetically be attributed to ChromAgar having a higher GC content compared to the IDEXX Colilert-18 method. Here, the IDEXX Colilert-18 method may operate under conditions more consistent with a minimum selective concentration (MSC) than a minimum inhibitory concentration (MIC) (10). At MSC-level exposures, even low-abundance resistant bacteria can be detected, providing a plausible explanation for the higher detection rate observed with Colilert-18 (10). Using an MSC-based approach could provide advantages in earlier detection of low levels of antimicrobial-resistant bacteria and therefore improved risk forecasting in environmental AR surveillance.

Of note, our results differ from those of a previous study which showed comparable performance of Colilert against MacConkey and Tryptone Bile X-glucuronide (TBX) agar for detecting ESBL *E. coli* (7). This difference may be attributed to methodological variations, such as differences in antibiotic concentration, selective media (TBX and MacConkey agars), and sample matrices used (soil and surface water).

Our cost analysis showed that the cost per sample of ESBL culture-based test ($13.63) was higher than for IDEXX Colilert-18 ($7.39). Similarly, the cost per ESBL *E. coli* detected was higher using ChromAgar ESBL culture ($35.05) compared to IDEXX Colilert-18 ($22.02). Reduced costs and personnel requirements offered by the IDEXX Colilert-18 method may enable environmental surveillance programs to be more scalable, especially in resource-limited settings (Table 2). Further cost-effective analysis of this method, including supply chain considerations and the use of willingness-to-pay thresholds, is needed. Given the fundamentally different quantification principles of the two methods (most probable numbers versus colony-forming units), direct comparison of *E. coli* concentrations was not methodologically appropriate, which represents a limitation of this study and confines interpretation to detection rather than bacterial load quantification.

**TABLE 2:** Comparison of costs between ChromAgar and ESBL Colilert methods^*a*^^,*b*,*c*^

Cost component	ChromAgar (USD)	Colilert (USD)
Staff time cost per test	8.83	1.52
Consumables cost per test	4.98	5.88
Total cost per test	13.63	7.39
Total costs (167 samples)	2,276.21	1,233.13
Cost per ESBL *E. coli* detected	35.05	9.72

^*a*^ The consumable costs are based on 2021 purchase prices.

^*b*^ All costs are in US dollar (USD).

^*c*^ Staff costs were based on 2021 average Lab Technician salary and estimated time per sample.

This study emphasizes the importance of urban marketplaces as potential hotspots for monitoring ESBL *E. coli* presence. Surveillance of these environments is likely to be a critical component of global efforts to track and mitigate AR. However, variations in the accuracy and cost of methods for detecting ESBL *E. coli* present challenges to effective global environmental surveillance efforts. Further research is needed to optimize and evaluate culture methods for the reliable recovery and identification of antimicrobial-resistant bacteria from environmental samples.

## SUPPLEMENTAL MATERIAL

Supplemental material spectrum.01695-25-s0001.docx Supplemental methods; Fig. S1.

Table S1 spectrum.01695-25-s0002.docx Comparison of prevalence of ESBL-producing *E. coli* in water stratified by market, season, and water source.

ASM does not own the copyrights to Supplemental Material that may be linked to, or accessed through, an article. The authors have granted ASM a non-exclusive, world-wide license to publish the Supplemental Material files. Please contact the corresponding author directly for reuse.
