# Supplementary material for: Evaluation of detection performance and cost-effectiveness of IDEXX Colilert-18 and ChromAgar for environmental surveillance of extended-spectrum beta-lactamase Escherichia coli in water samples from urban marketplaces in Blantyre, Malawi
Source: Microbiol Spectr. 2026 May 20;14(7):e01695-25. doi: 10.1128/spectrum.01695-25 (PMC13340141; doi:10.1128/spectrum.01695-25)
Supplement: Supplemental material — Supplemental methods; Fig. S1. [file spectrum.01695-25-s0001.docx]

**Methods for ESBL *E. coli* Detection in Water Samples Using Colilert®-18 and ChromAgar™**

**Contents**

[1. Introduction 1](#_Toc221539309)

[2. Sources of reagents and culture media 2](#_Toc221539310)

[3. ChromAgar Procedure 2](#_Toc221539311)

[3a. Materials 2](#_Toc221539312)

[3b. Storage conditions 2](#_Toc221539313)

[3c. Water sample processing 3](#_Toc221539314)

[3d. ESBL culture 3](#_Toc221539315)

[3e. Result/ Speciation 3](#_Toc221539316)

[4. Modified ESBL Colilert Procedure 4](#_Toc221539317)

[4a. Materials and equipment 4](#_Toc221539318)

[4b. Antibiotic preparation 5](#_Toc221539319)

[4c. ChromAgar ESBL Media preparation 5](#_Toc221539320)

[4d. Modified Colilert method 5](#_Toc221539321)

[4e. Results 5](#_Toc221539322)

[5. Technical and biological replicates 6](#_Toc221539323)

[6. Comparing the costs of the ESBL Colilert®-18 and ChromAgar™ methods 6](#_Toc221539324)

## **1. Introduction**

This document gives a description of the methods used for a study aimed at assessing ESBL E.coli detection abilities of Colilert®-18 and ChromAgar™ . These methods were applied for the culture of ESBL E. coli (ESBL -E) in water samples obtained from markets included in the Drivers for Resistance in Uganda and Malawi (DRUM) study.

Invasive infections caused by ESBL resistant Enterobacteriaceae including ESBL-E are a current healthcare burden in Malawi. Given the reduced availability of Carbapenems, many of these drug-resistant infections are locally untreatable. There has been prior interest to understand the drivers of antimicrobial resistance and transmission of these bacteria in environment samples. This needs excellent tools for environmental surveillance. Here we present some of the procedures for methods used for detecting ESBL -E in water.

## **2. Sources of reagents and culture media**

All the main reagents used for the said methods used in this study were obtained from standard commercial suppliers as detailed below:

1. CHROMagar™ ESBL was sourced from CHROMagar (Paris, France)
2. Colilert® from IDEXX Laboratories (Maine, USA)
3. Buffered Peptone Water (BPW) from Thermo Fisher Scientific (United Kingdom)
4. Ceftriaxone from Ningbo Nuobai Pharmaceutical Co. Ltd (China).

## **3. ChromAgar Procedure**

### **3a. Materials**

Nalgene plastic bottles

Vacuum filter

Filtration membrane

buffered-peptone water

ChromAgar ESBL agar plate

Sterile 1ul & 10ul Bacterial loops

Microbank storage vial with beads

1.8ml cryovials (premade with BPW/Glycerol)

Spot Indole Reagent

Whatman No. 1 filter paper

Gloves

Fine marker

### **3b. Storage conditions**

All reagents were stored as per the instructions of the manufacturer

### **3c. Water sample processing**

Water samples were collected in a sterile Nalgene plastic bottle and a filtered through a sterile 0.45µm cellulose ester gridded membrane on the vacuumed filter. A maximum volume of 500 mL water was filtered for each water sample**.** The filtration membrane was then aseptically placed into a sterile container with 30ml of BPW (pre-incubated at room temperature), using sterile tweezers and aerobically incubated at 37^o^C for 18-24 hours. After incubation a 200ul aliquot of the 30ml culture was processed for ESBL.

### **3d. ESBL culture**

Using a chromogenic agar plate, half streak plate was created using a 1µl microbiological loop of the incubated BPW (using 1 plate for 2 samples). The chromogenic agar plate was placed in an aerobic incubator at 37^o^C for 18-24 hours.

### **3e. Result/ Speciation**

Any growth was treated as a potential ESBL producer, but speciation of growth depended on colony color on chromogenic agar. Growth of dark pink to red colonies was sufficient speciation of ESBL E whereas metallic blue (+/- reddish halo) was considered ESBL KEC (*Klebsiella, Enterobacter, Citrobacter*) and further characterized with HRM-PCR.

*
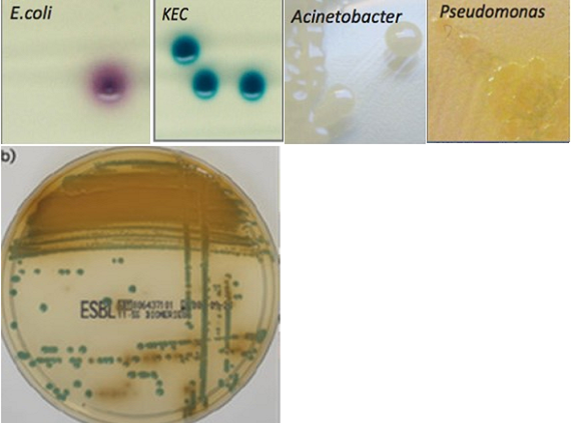
*

Figure 1; Interpretation of colony color on ChromAgar™ media

## **4. Modified ESBL Colilert Procedure**

This is an adapted version of the Colilert procedure to select for ESBL-E.

### **4a. Materials and equipment**

- P1000 Pipette
- P200 Pipette
- Quanti-Tray Sealer or Sealer Plus (IDEXX)
- Pipet-Aid pipette controller
- Biological Safety Cabinet
- Vortex mixer
- 35°C Incubator
- UV lamp
- Ceftriaxone sodium salt
- Sterile deionized water
- Syringe filter
- IDEXX Reagents: Colilert (IDEXX cat. WP2001)

### **4b. Antibiotic preparation**

Prepare a solution of 1 mg/mL ceftriaxone by adding 10 mg antibiotic to 10 mL sterile deionized water. Mix until the antibiotic is completely dissolved. Once the antibiotic has dissolved, the solution should be filter sterilized by passing it through a 0.22 μm syringe filter

### **4c. ChromAgar ESBL Media preparation**

Prepare ChromAgar ESBL plates as per the manufacturers protocol for use. Use these to validate ESBL E coli results of the modified Colilert method.

### **4d. Modified Colilert method**

1. Shake the water sample to mix then transfer 100ml of the sample into 4 different sterile 100ml vessels.
2. Add 100 microliters of the prepared antibiotic solution in 2 of the 100ml bottles and leave the other 2 without antibiotic solution. Label them accordingly with the sample name and ND (no drug) or CEF (ceftriaxone).
3. Add the contents of one Colilert reagent pack to each of the 100 mL water samples
4. Cap the vessel and shake until the reagent is dissolved
5. Pour the sample into a Quanti-Tray or Quanti-Tray/2000
6. Seal the tray with an IDEXX Quanti-Tray Sealer
7. Incubate the sealed tray at 37 ± 0.5°C for 18 to 24 hours

### **4e. Results**

The results are read under a biosafety cabinet using a UV lamp after 24 hours of incubating the quanti trays. Record the number of large and small wells for each treatment on a data sheet for total coliforms and ESBL *E coli.*

1. Yellow wells – Positive total coliforms
2. Wells that fluoresce under UV light – Positive ESBL E. coli

## **5. Technical and biological replicates**

To ensure reliability and reproducibility of ESBL E. coli detection, technical replicates were performed for both ChromAgar™ ESBL and the modified Colilert®-18 procedures. Each water sample was processed in duplicate for both methods. For the ChromAgar™ procedure, duplicate cultures were performed from the same enriched BPW sample. For the modified Colilert® method, each water sample was divided into duplicate vessels for both antibiotic-treated and non-treated samples.

## **6. Comparing the costs of the ESBL Colilert®-18 and ChromAgar™ methods**

First, the total cost for conducting Colilert test and culture was determined by calculating the costs for all activities and materials used during the surveillance of ESBLs. The costs included in the analysis comprised of the following: laboratory staff time (lab technician), cost of conducting the Colilert and Culture procedures and lab consumables required. Since the samples were collected from similar market sites, we assumed the costs of sampling to be equal for both tests as we did not include them in the final analysis. We also assumed any communication and utility bill costs to be equal for both tests. Time spent by staff on each activity (in hours) during the procedures was estimated/recorded per day. The respective salaries were then apportioned to each service according to the time spent on each test. The costs were added up to get total cost per test. We also calculated the total cost per ESBL E. coli detected. We also compared the cost for conducting one Colilert test and culture.
