# Supplementary material for: Evaluation of detection performance and cost-effectiveness of IDEXX Colilert-18 and ChromAgar for environmental surveillance of extended-spectrum beta-lactamase Escherichia coli in water samples from urban marketplaces in Blantyre, Malawi
Source: Microbiol Spectr. 2026 May 20;14(7):e01695-25. doi: 10.1128/spectrum.01695-25 (PMC13340141; doi:10.1128/spectrum.01695-25)
Supplement: Table S1 — Comparison of prevalence of ESBL-producing E. coli in water stratified by market, season, and water source. [file spectrum.01695-25-s0002.docx]

**Table 1: Comparison of prevalence of ESBL-producing E. coli in water stratified by market, season, and water source**

| **Factor** | **Groups** | **Total** | **Culture positive** | | **Colilert positive** | |
| --- | --- | --- | --- | --- | --- | --- |
|  |  |  | **n(%)** | **p-value** | **n(%)** | **p-value** |
| All | All | 167 | 65(38.9%) |  | 127(76%) |  |
|  |  |  |  |  |  |  |
| Market | Majiga | 47 | 21(44.7%) | 0.2 | 37(78.7%) | 0.55 |
|  | Ndirande | 40 | 13(32.5%) | 0.2 | 31(77.5%) |  |
|  | Ngumbe | 36 | 10(27.8%) | 0.2 | 24(66.7%) |  |
|  | Safarawo | 44 | 21(47.7%) | 0.2 | 35(79.5%) |  |
|  |  |  |  |  |  |  |
| Season | Rainy | 66 | 37(56.1%) | <0.001 | 53(80.3%) | 0.0914 |
|  | Cool-dry | 59 | 11(18.6%) |  | 39(66.1%) |  |
|  | Hot-dry | 42 | 17(40.5%) |  | 35(83.3%) |  |
|  |  |  |  |  |  |  |
| Water source | Drains water | 33 | 18(54.5%) | 0.00151 | 33(100%) | <0.001 |
|  | River water | 14 | 11(78.6%) |  | 11(78.6%) |  |
|  | Source water | 15 | 4(26.7%) |  | 4(26.7%) |  |
|  | Stored water | 95 | 30(31.6%) |  | 75(78.9%) |  |
